# Physical, psychological and social impact of school violence on children

**DOI:** 10.1186/s13052-019-0669-z

**Published:** 2019-06-27

**Authors:** Pietro Ferrara, Giulia Franceschini, Alberto Villani, Giovanni Corsello

**Affiliations:** 1 Institute of Pediatrics, Catholic University Medical School, Largo A. Gemelli, 8, 00168 Rome, Italy; 2 Service of Pediatrics, Campus Bio-Medico University, Rome, Italy; 3 Pediatric and Infectious Disease Unit, Bambino Gesù Children’s Hospital, IRCCS, Rome, Italy; 4 Institute of Pediatrics, University of Palermo, Palermo, Italy

**Keywords:** Violence, School, Children, Consequences

## Abstract

Violence against children includes all forms of violence against people under 18 years old whether perpetrated by parents or other caregivers, peers, partners, teacher or strangers. This is a public health, human rights, and social problem: levels of violence against children are frightfully high and it is estimated that up to 1 billion children aged 2–17 years, have experienced a type of violence. Very few studies provided physical violence perpetrated at school but it can have a physical impact, causing psychological distress, permanent physical disability and long-term physical or mental ill-health. Children who experienced any type of violence at school may develop reactive attachment disorder, modest physical inactivity, overweight or obesity, diabetes, smoking habits, heavy alcohol use, poor self-rated health, cancer, heart disease, and respiratory disease and other negative outcomes. Evidence from international studies clearly shows that nonviolent, positive discipline delivers better results, while any type of violence is associated with many negative one.

## Introduction

Violence against children is a public health, human rights, and social problem, with potentially devastating and costly consequences. [1] Globally, levels of violence against children are frightfully high and it is estimated that up to 1 billion children aged 2–17 years, have experienced physical, sexual, or emotional violence or neglect. [2]

Violence against children includes all forms of violence against people under 18 years old whether perpetrated by parents or other caregivers, peers, partners, or strangers. This wide definition of violence includes not only the more obvious violent acts of commission: at least one of six main types of interpersonal violence that tend to occur at different stages in a child’s development.

i. Maltreatment (including violent punishment): physical, sexual and psychological/emotional violence.
ii. Bullying (including cyber-bullying).
iii. Youth violence: concentrated among children and young adults aged 10–29 years, occurs most often in community settings between acquaintances and strangers.
iv. Intimate partner violence (or domestic violence): physical, sexual and emotional violence by an intimate partner or ex-partner.
v. Sexual violence: non-consensual completed or attempted sexual contact and acts of a sexual nature not involving contact.
vi. Emotional or psychological violence: restricting a child’s movements, denigration, ridicule, threats and intimidation, discrimination, rejection and other non-physical forms of hostile treatment. [3]

Violence may take place in homes, orphanages, residential care facilities, on the streets, in the workplace, in prisons and other place of detention and lastly at schools. Hardly any studies provided age-specific and sex-specific period prevalence estimates for physical violence perpetrated by teachers. [4]

Whereas children spend more time in the care of adults in schools and other places of learning than they do anywhere else outside of their homes; because of that violence that occurs at school should be under investigation for the physical, psychological and social problems arising from that. These consequences can be prompt, as well as latent, and can last for years after the initial violence.

In Italy a study conducted in the last 5 years reported 78 cases of school violence against children with a total of 156 investigated teachers (154 women and 2 males).

The number of teachers involved was various during those years: from 2016 to 2017 tripled, in 2018 a further 30% of episodes was reported. The phenomenon is still growing,also refers to the number the media’s alerts.

Geographical distribution analysis verified 23 cases in the North of Italy (30%), while 20 (25%) in the Center, and 35 (45%) in the South and in the Islands. Most cases 51 (65%) were found in provincial countries, while the remainder took place in urban centers 27 (35%).

The crime hypothesized was in 63 cases (81%) a maltreatment and 15 cases (19%) that of psychological violence. [5]

Plan International estimates that at least 246 million boys and girls suffer from school violence every year. [6]

### Type of violence at school

Violence in schools is one of the most visible forms of violence against children: it includes physical, psychological and sexual violence and bullying that are related to causes such as gender and social norms and wider structural and contextual factors such as income inequality, deprivation, marginalisation and conflict. [6]

Violence can be any form of physical aggression with intention to hurt(corporal punishment and physical bullying) by adults and other children. Corporal punishment is any punishment in which physical force is used and that is intended to cause some degree of pain or discomfort; it is often used to punish poor academic performance or to correct misbehaviour.

Psychological violence includes verbal and emotional abuse: isolating, rejecting, ignoring, insults, spreading rumours, making up lies, name calling, ridicule, humiliation and threats, and psychological punishment.

Psychological punishment are not physical but that humiliate, denigrate, scapegoat, threaten, scare or ridicule a child or adolescent. Sexual violence includes intimidation of a sexual nature, sexual harassment, unwanted touching, sexual coercion and rape, and it affects both girls and boys. Violence in schools creates insecurity and fear which harm the general school climate and infringe pupils’ right to learn in a safe, unthreatening environment.

Schools cannot fulfil their role as places of learning and socialisation if children are not in an environment free of violence. [7]

Violence, in particular physical one among learners, and physical violence perpetuated by teachers and other staff, can happen in sight of other learners for example, in playgrounds or classrooms or in the context of school sports. Teachers may also not recognise bullying or the codes, languages and practices children and adolescents use in harassing each other, and bullying that takes place out of their sight is difficult to identify. In some cases, teachers permit or engage in violent and bullying behaviour themselves. [8]

### Children and adolescents at risk of school violence

Violence against children is widespread and must be addressed to improve children’s health and well-being. [4] All children and adolescents could be at risk of school violence: but those who are vulnerable because of factors such poverty, social status associated with ethnicity, linguistic or cultural differences and migration or displacement, and disabilities, who are orphans or from households affected by HIV, may be more likely to be targeted. [9]

Punishment by teachers may be more likely to target children and adolescents from stigmatised and marginalised populations, for example, refugee and migrant children may be punished for not being able to speak the language of instruction, and the UN Study on Violence against Children notes that in India, higher caste teachers may be more likely to denigrate and humiliate children from lower castes. [10, 11]

Boys, especially younger children, are more likely to be punished physically at school, while the percentage of children verbally punished is pretty much equal for boys and girls. [12]

### Where school violence occurs

School violence can occur inside and outside the classroom, around schools, on the way to and from school. Violence, in particular physical violence among learners, and physical violence perpetuated by teachers and other staff, can happen in sight of other learners for example, in playgrounds or classrooms or in the context of school sports. [13]

All forms of violence in schools infringe the fundamental right to education and unsafe.

learning environments reduce the quality of education for all learners.

### Physical impact of school violence on children

Violence in school can have a physical impact and it can cause psychological distress, permanent physical disability and long-term physical or mental ill-health. Physical impacts are the most obvious and may include mild or serious wounds, bruises, fractures, and deaths by homicide or suicide. A number of studies have shown correlations between corporal punishment and poor mental health. [14] While most have focused on corporal punishment within families, some have focused on corporal punishment in schools with a social impacts invariably negative. Victims of corporal punishment are likely to become passive and overly cautious, and to fear free expression of their ideas and feelings while, at the same time, they may become perpetrators of psychological violence. Children who are physically punished are less likely than other children to internalise moral values and they are less inclined to resist temptation, to engage in altruistic behaviour, to empathise with others or to exercise moral judgement of any kind. They are more inclined to develop disorderly and aggressive conduct such as hitting their siblings, parents, schoolmates and boyfriends or girlfriends. And they may become adults who are prone to punishment against their own children, and so pass on the habits of violence. [15]

### Reactive attachment disorder and other social problems

Children who experienced any type of violence at school may develop reactive attachment disorder that is classified by The Diagnostic and Statistical Manual 5th Edition (DSM-5) as a trauma- and stressor-related condition of early childhood caused by social neglect and maltreatment. Affected children have difficulty forming emotional attachments to others, show a decreased ability to experience positive emotion, cannot seek or accept physical or emotional closeness, and may react violently when held, cuddled, or comforted. Behaviourally, affected children are unpredictable, difficult to console, and difficult to discipline. They have a strong desire to control their environment and make their own decisions. Changes in routine, attempts to control, or unsolicited invitations to comfort may elicit rage, violence, or self-injurious behaviour. In the classroom, these challenges inhibit the acquisition of core academic skills and lead to rejection from teachers and peers alike. Abuse in childhood has been correlated with difficulties in working memory and executive functioning, while severe neglect is associated with underdevelopment of the left cerebral hemisphere and the hippocampus. Children are more likely than their neuro-typical peers to engage in high-risk sexual behaviour, substance abuse, have an involvement with the legal system, and experience incarceration [14–17] Children may respond to inter-linkages with aggression, fear, defiance, or rage; they develop a negative self-schema, and experience somatic symptoms of distress. Psychomotor restlessness is common, as is hyperactivity and stereotypic movements, such as hand flapping or rocking. It is confirmed an increased risk of anxiety, depression, hyperactivity, and reduces frustration tolerance. [17, 18]

Researches identifies the harmful effects that adverse childhood experiences (ACEs) occurring during childhood or adolescence; eg, child maltreatment or exposure to domestic violence) have on health throughout life.

Exposure to ACEs is associated with a host of harmful outcomes, including increased risk for cancer. [19, 20] Individuals with at least four ACEs were at increased risk of all health outcomes compared with individuals with no ACEs. Associations were weak or modest for physical inactivity, overweight or obesity, and diabetes (ORs of less than two); moderate for smoking, heavy alcohol use, poor self-rated health, cancer, heart disease, and respiratory disease (ORs of two to three), strong for sexual risk taking, mental ill health, and problematic alcohol use (ORs of more than three to six), and strongest for problematic drug use and interpersonal and self-directed violence (ORs of more than seven). [20] Psychosocial adversity in childhood (e.g. abuse) is demonstrated to be related to DNA methylation age acceleration: suggesting a potential mechanism linking violence with adverse outcomes. [21] Evidence suggests that adverse experiences in childhood (particularly exposure to multiple adversities involving hostility and threat), in some people, contribute to the onset of psychotic experiences and psychotic disorders are associated with psychosis too. [22] Recent work demonstrates severity of child maltreatment, as well as presence of childhood physical and sexual abuse, are associated with adolescent cannabis use (any use versus non-use) and heavy cannabis use during adolescence. Research has demonstrated that experiences of childhood maltreatment are prevalent in the life histories of youth with substance use problems; however, most of this research has focused on sexual or physical abuse. [23, 24]

The educational effects on victims of school violence are significant. Violence at the hands of teachers or other students may make children and adolescents afraid to go to school and interfere with their ability to concentrate in class or participate in school activities. It can also have similar effects on bystanders. The consequences include missing classes, avoiding school activities, playing truant or dropping out of school altogether. This in turn has an adverse impact on academic achievement and attainment and on future education and employment prospects. Children and adolescents who are victims of violence may achieve lower grades and may be less likely to anticipate going on to higher education. Analyses of international learning assessments highlight the impact of violence and bullying on learning outcomes. These analyses clearly show that it is reduced students’ achievement in key subjects such as mathematics and other studies have also documented the negative impact of school violence and bullying on educational performance. [25–28]

## Conclusion

Violence against children is a significant cause of physical problems, psychological distress, permanent physical disability and long-term physical or mental ill-health.

Governments should provide that the law provides children and protects their human dignity. Evidence from international studies clearly shows that nonviolent, positive discipline delivers better results, while any type of violence is associated with many bad outcomes. The adoption of the most effective teaching approach across the education system, by supporting teachers to develop non-violent, positive discipline strategies could be the right way to step closer to realising children’s right to protection from all forms of violence in all settings, included the school.

## Data Availability

The datasets used and/or analysed during the current study are available from the corresponding author on reasonable request.

## Abbreviations

(ACEs): Adverse childhood experiences
DSM-5: Diagnostic and Statistical Manual 5th Edition
